# 4D Segmentation of Brain MR Images with Constrained Cortical Thickness Variation

**DOI:** 10.1371/journal.pone.0064207

**Published:** 2013-07-02

**Authors:** Li Wang, Feng Shi, Gang Li, Dinggang Shen

**Affiliations:** 1 Department of Radiology and BRIC, University of North Carolina at Chapel Hill, Chapel Hill, North Carolina, United States of America; 2 Department of Brain and Cognitive Engineering, Korea University, Seoul, Korea; University of Minnesota, United States of America

## Abstract

Segmentation of brain MR images plays an important role in longitudinal investigation of developmental, aging, disease progression changes in the cerebral cortex. However, most existing brain segmentation methods consider multiple time-point images individually and thus cannot achieve longitudinal consistency. For example, cortical thickness measured from the segmented image will contain unnecessary temporal variations, which will affect the time related change pattern and eventually reduce the statistical power of analysis. In this paper, we propose a 4D segmentation framework for the adult brain MR images with the constraint of cortical thickness variations. Specifically, we utilize local intensity information to address the intensity inhomogeneity, spatial cortical thickness constraint to maintain the cortical thickness being within a reasonable range, and temporal cortical thickness variation constraint in neighboring time-points to suppress the artificial variations. The proposed method has been tested on BLSA dataset and ADNI dataset with promising results. Both qualitative and quantitative experimental results demonstrate the advantage of the proposed method, in comparison to other state-of-the-art 4D segmentation methods.

## Introduction

With the rapid development of MR imaging technology and its widespread use, large number of MR images are obtained for clinical studies. Longitudinal studies, aiming to capture the time related brain changes, are becoming more common by acquiring multiple time-point images from each subject. It has many applications, e.g., mapping early brain development, aging, and tracking the progression and onset of neurodegenerative diseases such as Alzheimers disease (AD). From image processing point of view, brain segmentation is a fundamental step to label brain into anatomically meaningful tissues such as gray matter (GM), white matter (WM), and cerebrospinal fluid (CSF). Many algorithms have been proposed for this purpose, such as SPM8 [1], FAST [2], FANTASM [3], PVC [4], and CRUISE [5]. However, segmenting brain image independently will introduce random errors for the results of different time-points. For example, the resulting cortical thickness measured from the segmented image will contain unnecessary temporal variations, which will affect the time related change pattern and eventually reduce the statistical power of analysis. To this end, several 4D segmentation methods were proposed in recent years to address this problem by including the temporal constraint between time-points in the segmentation process [6]–[10]. In [6], the authors proposed a temporally consistent and spatially adaptive longitudinal MR brain image segmentation algorithm based on FANTASM, referred to as CLASSIC, which aims at obtaining accurate measurements of rates of change of regional and global brain volumes from serial MR images. It iteratively performs two steps: (i) first jointly segments a series of 3D images using a 4D image-adaptive clustering algorithm based on the current estimate of the longitudinal deformations in the image series; (ii) then refines these longitudinal deformations using 4D elastic warping algorithm. However, CLASSIC works voxel by voxel, and accumulated subtle errors in brain tissue segmentation may largely affect the subsequent cortical surface construction as well as the calculation of cortical thickness. Recently, a longitudinal processing pipeline was proposed in FreeSurfer [7]. In this pipeline, a group-mean image is firstly generated by averaging from the rigidly-aligned longitudinal images of a subject. The cortical surfaces of the group-mean image are then used as initialization for each longitudinal image. Finally, the cortical surfaces at each time point are deformed to achieve longitudinal cortical surface reconstruction.

In addition, the measurement of cortical thickness is of great interest in studying normal brain development, aging, and a wide variety of neurodegenerative and psychiatric disorders. Neuroscience studies have suggested that various diseases such as AIDS or AD may affect the cortical thickness [11]. Thus, by accurate measurement of cortical thickness, one hopes to have early detection for certain brain diseases for possibly early treatment. Accordingly, based on the fact that the thickness of the human cerebral cortex varies smoothly over the whole cortex, ranging between 1 and 5 mm [12]–[14], Zeng et al. [15] first introduced the idea of using coupled level sets for segmentation of the brain cortex. The ideas introduced by Zeng et al. were later extended by Goldenberg et al. who proposed a fast variational geometric approach for cortex segmentation [16]. Although the cortical thickness constraint were utilized in these methods, they were utilized only in the spatial domain, not for the longitudinal images. To date, few algorithms have considered the constraint of the changes of cortical thickness in the longitudinal studies. For example, cortical thinning occurs by middle age and spans widespread across cortical regions, including primary as well as association cortex [17]. The majority of the cortical mantle showed thinning rate of at least 0.01 mm/decade [17]. This inspires us to temporally constrain the change of cortical thickness in our longitudinal segmentation algorithm, which is biologically meaningful.

We previously proposed a brain segmentation method for infant images [18]. The main idea is to use the segmentation result from late-time-point image (with better image contrast in more matured brain) as prior to guide the segmentation task on early-time-point images. There are two limitations in the previous work. First, the temporal guidance is one-way from late-time-point to early time-point. Second, only two time-points were included in this framework. For example, even if there are more late-time-point images, only one can be selected and contribute to the segmentation of early-time-point image. In this paper, we propose a fully 4D brain segmentation method to address the two limitations. First, we update the framework with 4D formulation, so temporal guidance can be collected from all time-points. Second, images at all time-points are involved to the segmentation process. Moreover, we introduce a cortical thickness constraint in neighboring time-points to suppress the artificial variations. In the next section, we discuss the methodology details of the proposed method. Experiments are followed to evaluate the performance of proposed method, in comparisons to manual ground truth and other 4D methods.

## Materials and Methods

### Overview of the Proposed Method

**Figure 1:**
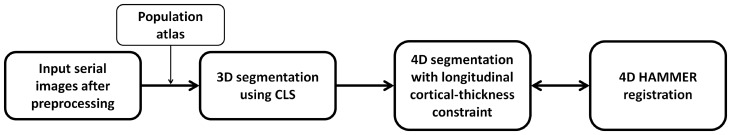
The proposed framework for the 4D brain segmentation.

An overview of the proposed framework is shown in Fig. 1. We first use the coupled level sets (CLS) [19] to initially segment the all time-point images separately with a population atlas [20]. These 3D segmentation results are then input into the 4D segmentation and registration components.

In the following, we will detail our 4D segmentation and registration components. The proposed energy function for this 4D component consists of three terms, i.e., local data fitting term, spatial cortical thickness constraint term, and temporal cortical-thickness constraint term. Since details of the local data fitting term and spatial cortical thickness constraint term can be found in our previous work [18], we will briefly overview these two terms and mainly focus on the temporal cortical thickness change constraint term in the following subsections.

### Local Data Fitting Term

To effectively exploit information on local intensities, we need to accurately estimate the distribution of local intensities. For each voxel 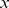 in the image domain 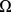, we can define a spherical neighborhood with a small radius 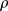, such as 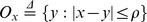. Let 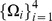 denote four different regions, i.e., white matter (WM), grey matter (GM), cerebrospinal fluid (CSF) and the background. For 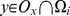, such as in the case that the neighborhood 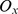 of the voxel 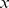 sitting within the *i*-th image region 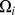, its intensity distribution model can be parameterized by a Gaussian model 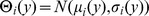. To accurately measure the probability of image intensity 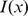 at voxel 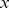 belonging to 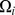, we estimate it from all the intensity distribution models in the neighborhood of voxel 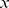, i.e., 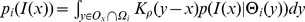, instead of using a single model 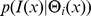 as in the conversional methods, where 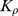 is a Gaussian kernel with scale 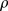 to control the size of the neighborhood [21]. Since 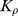 controls the size of the neighborhood 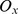, the probability 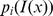 can be further simplified as 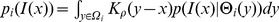. Taking a logarithm, the maximization of probability 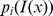 can be converted to the minimization of the following energy, denoted by
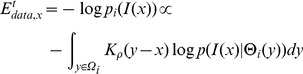
(1)


For all the voxels 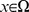, we can define a local intensity fitting energy function as the following double integral:
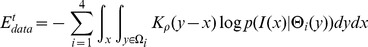
(2)


In this paper, the level set function takes negative values outside of the zero-level-set and positive values inside of the zero-level-set. Denoting three level set functions at time-point 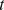 as 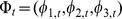 and with the help of the Heaviside function 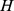, the regions corresponding to WM, GM, CSF and the background, i.e., 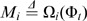, 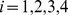, can be defined respectively as 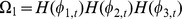, 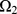 = 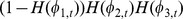, 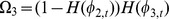, and 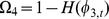. Additionally, due to large overlap among the tissue distributions, it is helpful to use spatial prior 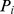 for guiding the segmentation. Therefore, Eq. (2) can be reformulated as,
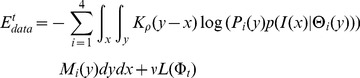
(3)where 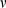 is a weighting constant (we set 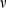 = 0.5 in this paper), and the second term 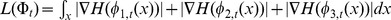 is the length term to maintain a smooth contour/surface during the evolution. A population atlas was utilized as the spatial prior 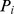 to segment the all time-point images.

### Spatial Cortical Thickness Constraint Term

As pointed out in [15], [16], [22]–[24], the variation of regional cortical thickness is smooth, and therefore can be used as a constraint to guide cortical surface reconstruction. To utilize this information, we designed a cortical thickness constraint term to constrain the distance of zeros level surfaces of 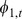 and 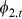 (which represents the inner and outer cortical boundaries) within a predefined range 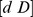, where 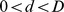 (in this paper, 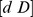 is set as [1 6.5]mm). Thus, for each point on the outer cortical surface, we compute its closest point on the inner cortical surface and define their Euclidean distance as the cortical thickness of the point. Note that the level set function is a signed distance function, therefore, for any point on the outer cortical surface, the absolute value of 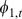 at this point is simply the closest Euclidean distance from the point to the inner cortical surface (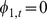). The spatial constraint term [18] is defined for 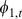,
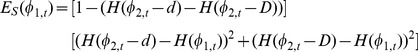
(4)


In a similar way, we can define a distance constraint term for 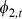,
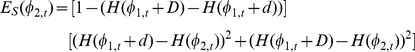
(5)


### Temporal Cortical Thickness Variation Constraint Term

**Figure 2:**
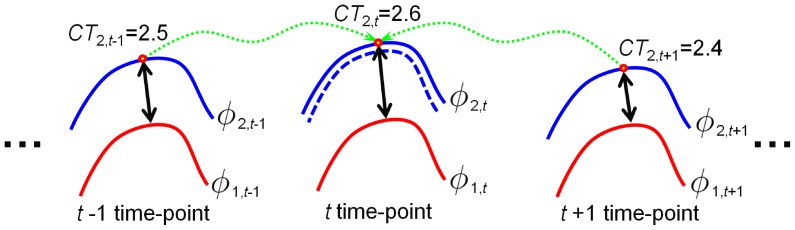
Illustration of temporal cortical thickness variation constraint. The solid red (or blue) curves are the zero-level surface of 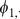 (or 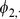). 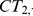 is the cortical thickness measured from the inner surfaces (red curves). The dashed green arrows denote the registration operation to warp the corresponding thickness from the temporal neighborhoods to the current time-point. The dashed blue curves in the middle is the reasonable surface determined after measuring the cortical thickness difference between current time-point and two neighboring time-points.

It is known that the cortical thickness is changing slowly and smoothly in the life time span [17]. Measurement of longitudinal cortical thickness change is highly important for analysis of diseases related with cortical thickness change, such as Alzheimer’s disease. With the help of the 4D HAMMER registration [25], we can identify the anatomical correspondence between different time-points. Recall that the inner and outer surfaces are the zero-level surfaces of 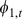 and 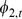. Thus, for any point on the outer cortical surface (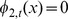), the absolute value of 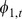 at this point is simply the distance from the point to the inner cortical surface (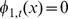). Let 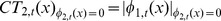 be the thickness measured from the zero-level surface of 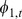, and 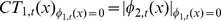 be the cortical thickness measured from the zero-level surface of 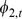. As shown in Fig. 2, with the help of 4D registration algorithm [25], we can compare the cortical thickness of current time-point with the corresponding cortical thickness of the other neighboring time-points. Let 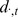 be the summation of the thickness differences between current time-point 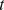 and the neighboring time-points.

For the level set 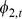, its temporal cortical thickness variation constraint term is defined as,
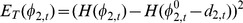
(6)

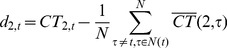
(7)where 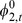 is the initialization of 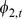 before the surface evolution, 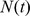 is the temporal neighborhood around the current time-point 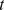, 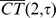 is the corresponding cortical thickness from the time-point 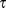. In this paper, we use the previous time-point 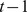 and the next time-point 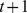 to calculate 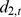, i.e., the immediate temporal neighbors. For example, in Fig. 2, for the red point in the outer curve (shown in blue color), the cortical thickness difference is 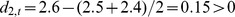, which means that the thickness of current point is thicker than the mean thickness of the temporal neighbors. Therefore, the cortical thickness variation constraint term 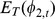 has the tendency to deflate the zero-level surface of 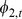, i.e., to the dashed blue curve, and hence to decrease the cortical thickness to suppress the artificial variations.

Similarly, we can define a cortical thickness variation constraint term for 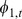,
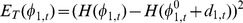
(8)

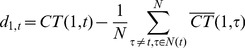
(9)


Therefore, the final energy function for the segmentation of longitudinal brain MR images can be defined as below, which combines the local intensity information, spatial cortical thickness constraint, and temporal cortical thickness variation constraint:
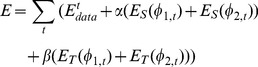
(10)where 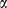 and 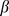 are the blending parameters. To effectively minimize this energy with respect to 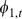 and 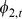, we can convert it as follows,



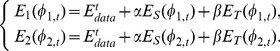
(11)By calculus of variations, the minimization of the energy function 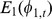 and 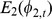 with respect to 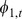 and 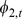 are achieved by solving the gradient descent flow equations as follows,
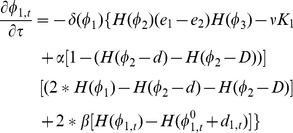
(12)

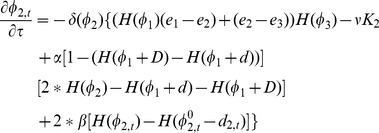
(13)where 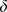 is the Dirac delta function, computing the derivative of the Heaviside function *H*. 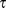 denotes the evolution time, in contrast to the physical time *t*, 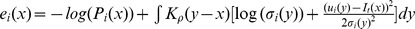 and 
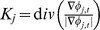
. The 4D segmentation and registration are performed alternately, i.e., after each step of evolution of Eq. (10), the 4D registration [25] is performed to derive a new cortical thickness differences between time-points to guide the next evolution of 4D segmentation.

## Experimental Results and Analysis

The preprocessing of the input longitudinal images includes the following steps: (1) intensity correction of each image using N3 [26]; (2) to avoid bias, the input serial images are rigidly aligned onto an atlas space and the group-mean image can be constructed by averaging all rigidly aligned images; (3) skull stripping [27] and removing the cerebellum using in-house tools on the group-mean image; (4) warping the brain mask of the group-mean image back to the each time-point image space based on the inverted transform matrix and then removing the non-brain using the warped brain mask.

In our experiments, we set the allowable cortical thickness to [1], [6], [5]mm, the length term 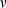 = 0.5, the weight parameter for the spatial cortical thickness term 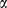 = 1, and 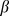 = 0.5 for the temporal cortical thickness term. The functions 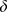 and 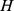 are regularized as in [28]. The level set functions are reinitialized as the signed distance functions at every iteration by using the fast marching method [29]. To measure the overlap rate between the two segmentations 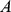 and *B*, we employ the Dice ratio (DR), defined as 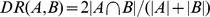. DR ranges from 0 to 1, corresponding to the worst and the best agreement between labels of two segmentations.

### Results on Simulated Data

**Figure 3:**
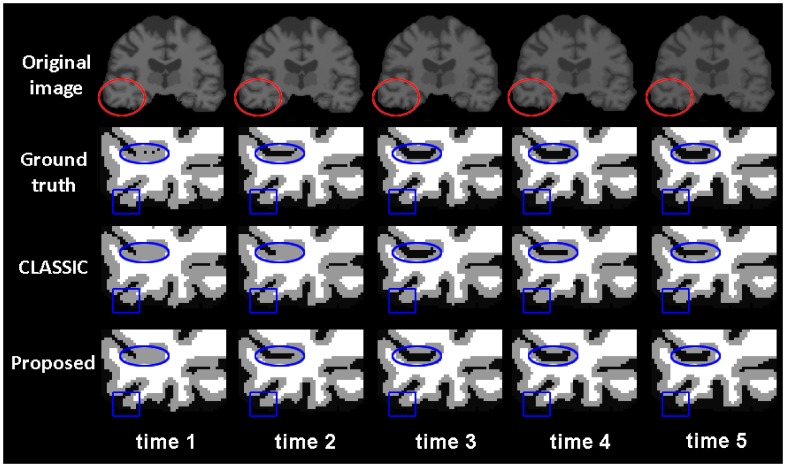
Rows from top to bottom show the simulated intensity images, ground-truth segmentation, and segmentation results by CLASSIC and the proposed method, respectively.

**Figure 4:**
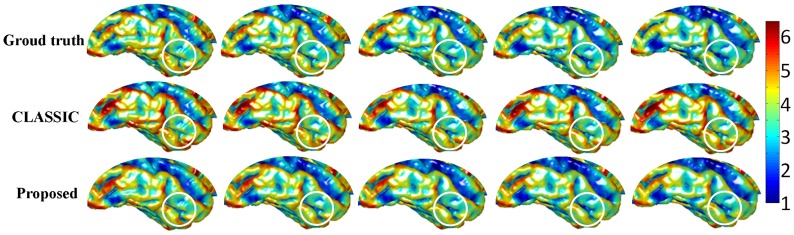
Rows from top to bottom show the cortical thickness maps of ground truth (1st row), thickness maps by CLASSIC (2nd row), and thickness maps by the proposed method (3rd row).

**Figure 5:**
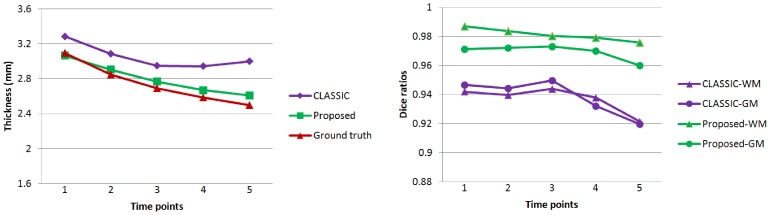
Comparison of cortical thickness and tissue overlap with the ground truth by CLASSIC and the proposed method. *Left*: The thickness maps. *Right*: Dice ratios of CLASSIC and the proposed method for WM and GM, respectively.

To generate simulated images with longitudinal deformations, we used Atrophy Simulation Package (http://www.rad.upenn.edu/sbia/projects/atrophy_simulation.html), which can simulate the atrophy by matching the Jacobian of the simulated deformation to the desired volumetric changes, subject to smoothness and topology preserving constraints employed in the algorithm [30]. The amount of atrophy can be defined by the shrinkage rate, 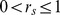. For example, 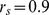 implies a 10% atrophy within the spherical area. In this paper, we set 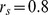. By using this package, we can simulate a longitudinal segmented images with 5 time-points. To simulate the decrease of intensity/contrast in aging, we set initial intensities of CSF, GM and WM of the 1st time-point image with means of [25,85,105], which were computed from the real images from our datasets. For the following 4 time-points, CSF has constant intensity as 25, while GM and WM are gradually declined with 2 and 4, respectively. We then added some Gaussian noise to each image and used a Gaussian kernel to smooth the image to simulate the partial volume effect. Fig. 3 shows the simulated intensity images, ground-truth segmentations, and corresponding segmentation results using CLASSIC and the proposed method, respectively. The red circles denotes the spherical area within which the atrophy and intensity/contrast decrease were simulated. The ground truth of cortical thickness maps are shown in the first row of Fig. 4, in which the atrophy can be clearly visualized (see the circled regions). The cortical thickness maps by CLASSIC and the proposed method are shown in the 2nd and 3rd row of the Fig. 4, respectively. By visual comparison, we can find that the thickness maps by CLASSIC are not temporally consistent. For example, as shown in Fig. 5, the thickness is increased at the time-point 4 and 5 for the CLASSIC result, which is generally impossible for the elderly brains. On the other hand, our results are much more similar with the results of the ground truth. The Dice ratios computed by comparison with the ground-truth segmentation, shown in the right of Fig. 5, also demonstrate that the proposed method achieves more accurate segmentation results.

### Results on the BLSA Dataset

**Figure 6:**
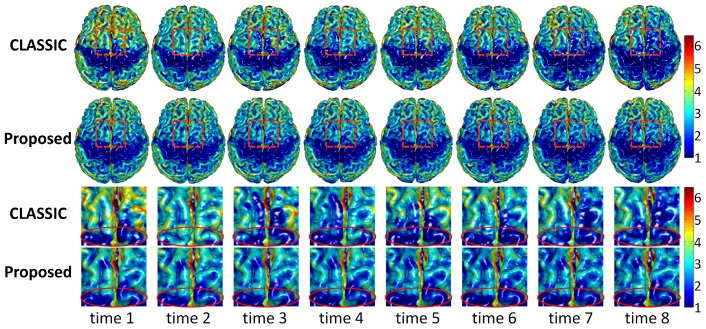
Cortical thickness maps derived by CLASSIC (the 1st row) and the proposed method (the 2nd row) on a randomly selected subject from the BLSA dataset. The last two rows show the zoomed views of the first two rows.

**Figure 7:**
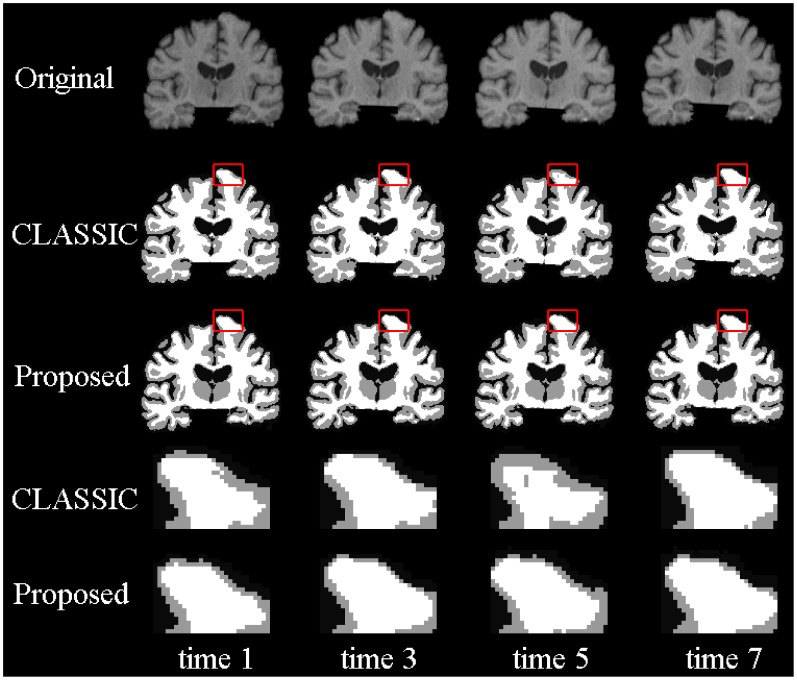
The first row shows the original intensity images, and the next two rows show the segmentation results by CLASSIC and the proposed method, respectively. The last two rows show the zoomed views of the 2nd and 3rd rows.

**Figure 8:**
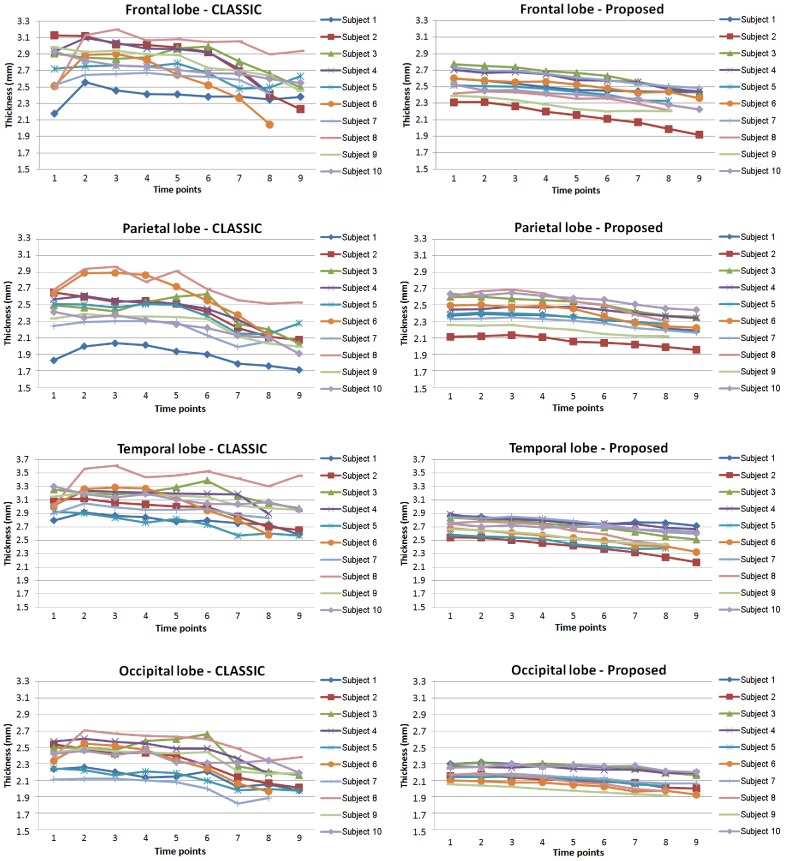
The average cortical thicknesses on 4 lobes of 10 elderly subjects from the BLSA dataset measured by CLASSIC (left column) and our proposed method (right column). One curve indicates for one subject.

To validate our proposed method, we apply our method to longitudinal brain MR images of 10 elderly subjects from the Baltimore Longitudinal Study of Aging (BLSA) dataset [31]. In these 10 subjects, each subject has been successively scanned 8 or 9 times, with the interval of about 1 year. Thickness maps by CLASSIC and the proposed method of a randomly selected subject are shown in Fig. 6. From the zoomed views in the two lower rows, we can clearly see that the cortical thickness changes dramatically between neighboring time-points by CLASSIC. For example, the thickness in the lower part of 2nd time-point (circled regions) is even much thicker than the thickness of the 1st time-point, which is generally unrealistic in aging brains. The inconsistency can also be observed from the segmentation results in Fig. 7. For the zoomed views in the two lower rows, the segmentation result of 5th time-point are quite inconsistent with the results of the other time-points. The average thickness on 4 lobes by CLASSIC and the proposed method are also shown in Fig. 8. As we can see, the average cortical thickness declines along the time by our proposed method, while the results by CLASSIC appears bumpy.

### Results on the ADNI Dataset

**Figure 9:**
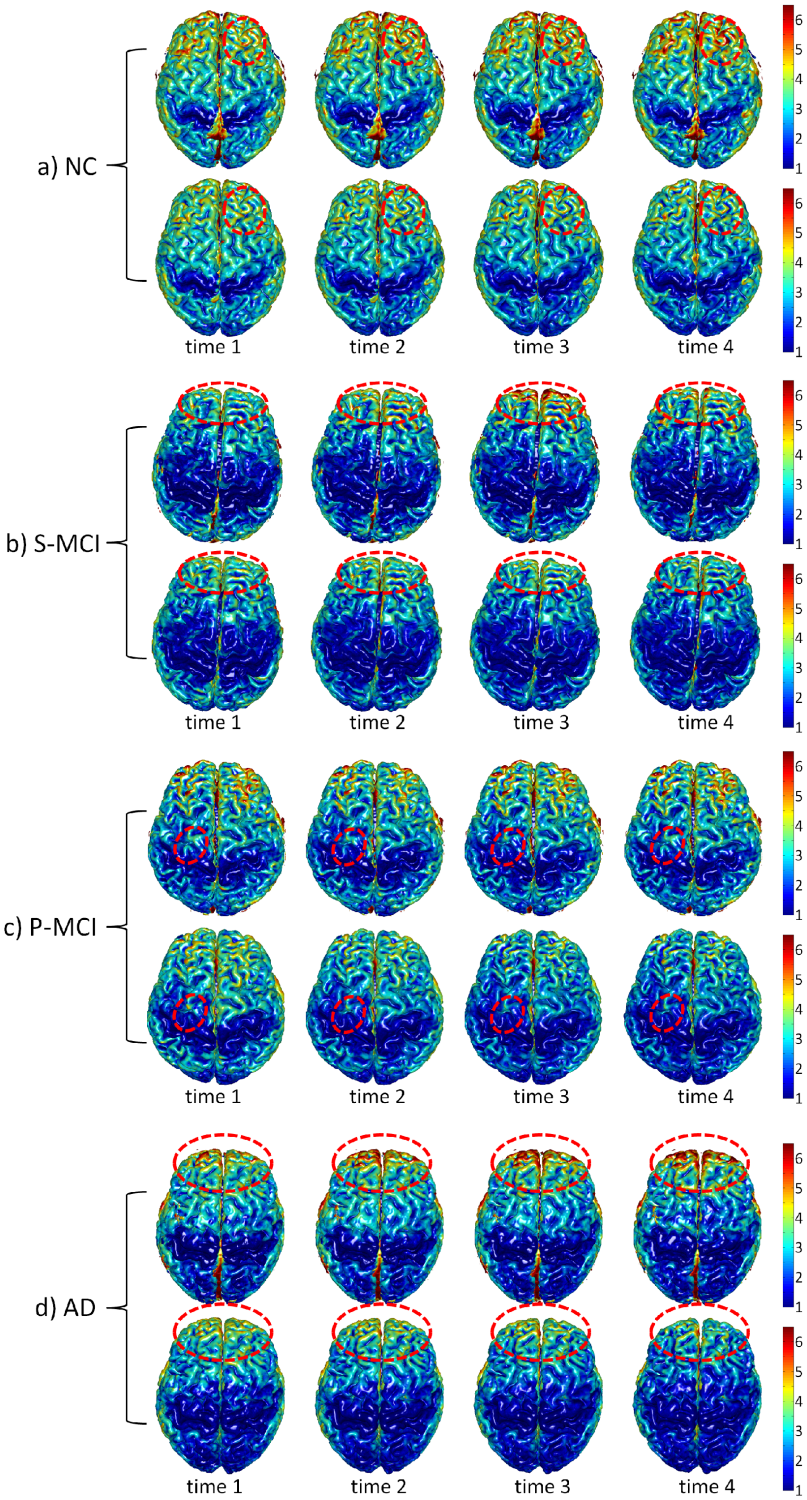
Cortical thickness maps derived by CLASSIC and the proposed method on the 4 reprehensive subjects from a) NC, b) S-MCI, c) P-MCI, and d) AD groups. In each group, the upper row shows the results of CLASSIC and the lower row shows the proposed results. Circles indicate the region with dramatic thickness changes by CLASSIC, while consistent measurement achieved by our proposed method.

**Figure 10:**
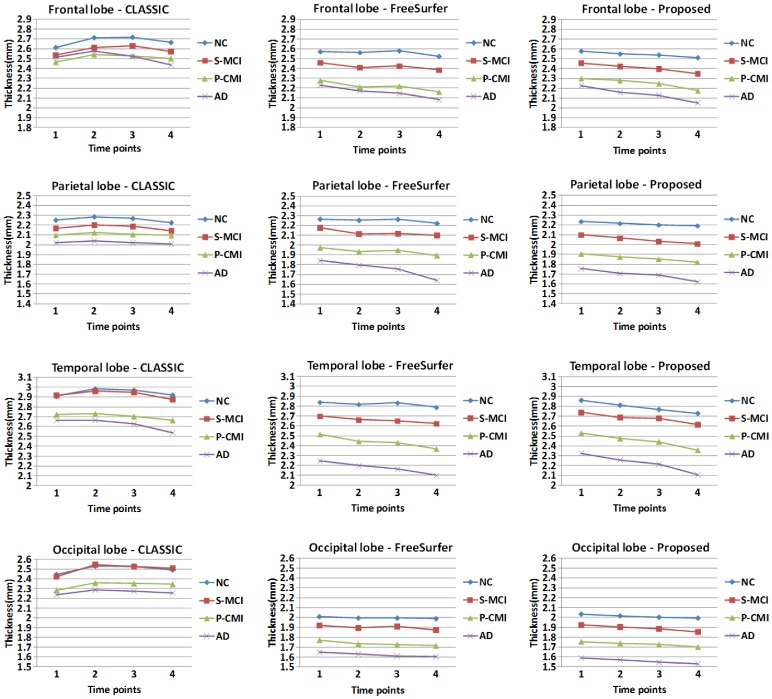
The average cortical thickness on 4 lobes derived by CLASSIC (the left column), the FreeSurfer (the middle column) and the proposed method (the right column) on all subjects from NC, S-MCI, P-MCI and AD groups.

To further validate our proposed method, we applied the method to the ADNI dataset with four groups of subjects, including the normal control (NC), stable mild cognitive impairment (S-MCI), progressive mild cognitive impairment (P-MCI), and AD groups, in which each group contains about 37 subjects with 4 time points in 24 months. Thickness maps of four representative subjects from each group are shown in Fig. 9. As we can see, the thickness maps by CLASSIC (upper row in each panel of Fig. 9) are bumpy temporally, especially in the regions indicated by the red circles. In contrast, the results by the proposed method (lower row in each panel of Fig. 9) are much more consistent along time than CLASSIC. We also calculate the mean cortical thickness in the four lobes from all subjects, as shown in the Fig. 10. The thickness by CLASSIC (the 1st column) at the second time-point even became thicker than that at the baseline. While for the results by the proposed method (the 3rd column), the decline trend of cortical thickness is apparent. The largest decreasing trend of cortical thickness is shown in the temporal lobes of the AD group, which is consistent to the findings in the literature [32], [33]. Overall, the NC group has the largest average cortical thickness at the baseline and also has the slowest longitudinal thickness decline trend. While, the AD group has the smallest average cortical thickness at the baseline and the fastest longitudinal thickness decline trend. The longitudinal cortical thicknesses of S-MCI and P-MCI groups are in-between that of the NC and AD groups. And the S-MCI group is relatively close to the NC group, and the P-MCI group is relatively close to the AD group, in terms of both the baseline thickness and longitudinal thickness decline trend [8].

### Comparison with FreeSurfer

**Figure 11:**
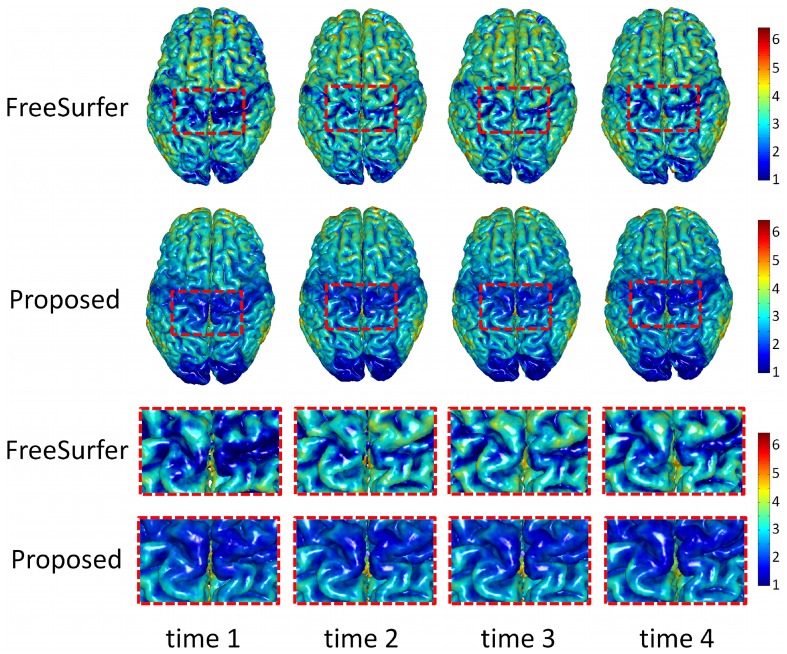
Cortical thickness maps derived by FreeSurfer (the 1st row) and the proposed method (the 2nd row) on a randomly selected normal subject from the ADNI dataset. Regions indicated by the dotted curves show dramatic longitudinal changes of cortical thickness by FreeSurfer, while much consistent results by the proposed method.

**Figure 12:**
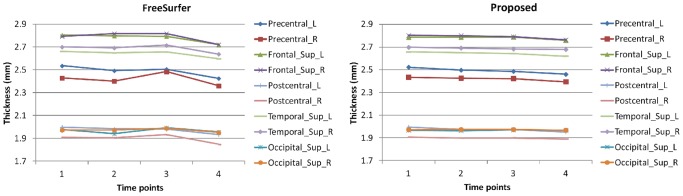
Average cortical thickness in 10 representative small cortical regions (ROIs) on all NC subjects. These 10 ROIs include the left (L) and right (R) parts of *Precentral*, *Frontal Sup*, *Postcentral*, *Temporal Sup*, and *Occipital Sup* regions, where *Sup* denotes superior gyrus.

In this section, we make comparisons with the recent longitudinal processing pipeline developed in FreeSurfer [7]. In the FreeSurfer pipeline, a group-mean or group-median image is firstly generated by averaging from the rigidly-aligned longitudinal images of a subject. The cortical surfaces of the group-mean image/group-median are then used as initialization for each longitudinal image. Finally, the cortical surfaces at each time point are separately deformed to achieve longitudinal cortical surface reconstruction. Thickness maps by FreeSurfer and the proposed method of a randomly selected subject are shown in Fig. 11. Although FreeSurfer can guarantee that the reconstructed longitudinal cortical surfaces at different time points have exactly the same triangular mesh configuration and topology with the cortical surfaces of the group-mean/group-median image, no temporal constraint is imposed in the FreeSurfer, thus the temporal trajectories of attributes (such as positions and cortical thicknesses) of vertices on longitudinal cortical surfaces are generally bumpy [8]. For example, from the zoomed views in the two lower rows, we can clearly see that the cortical thickness changes dramatically from the 1st time-point to the 2nd time-point by FreeSurfer. The average thickness on all subjects from NC, S-MCI, P-MCI, and AD groups is shown in the middle column of Fig. 10 with comparison with the proposed method, from which we can also find the thickness maps by FreeSurfer are bumpy temporally, although the overall descend trend is similar with the proposed method. Taking the NC group for an example, the thickness of the temporal lobe at the 3rd time-point is even larger than the 2nd time-point point in the results by FreeSurfer. Compared with the results by FreeSurfer, the thickness measured by the proposed method is more consistent. To better show the advantage of the proposed method, we further parcellate the cerebral cortex into 78 cortical regions instead of 4 lobes by employing the Automated Anatomical Labeling (AAL) template [34] and calculate the average thickness in these small cortical regions (ROIs). Fig. 12 shows the average cortical thickness in 10 representative ROIs on all NC subjects. These 10 ROIs include the left (L) and right (R) parts of *Precentral*, *Frontal Sup*, *Postcentral*, *Temporal Sup*, and *Occipital Sup* regions, where *Sup* denotes superior gyrus. It can be clearly seen that the thickness by the FreeSurfer without temporal constraint is bumpy, while our result is much more temporally consistent.

## Discussion

In this paper, we have proposed a novel 4D brain segmentation framework with applications in the elderly brain MR images. The temporal guidance is collected from all time-points and images at all time-points are involved to the segmentation process. Moreover, a cortical thickness constraint in neighboring time-points was proposed to suppress the artificial variations.

The average total computation cost is around 5.5 hours for the segmentation of a serial images with 4–5 time-points in MATLAB environment on a PC with 2.5 GHz Pentium4 processor. In this computational time, 0.5 hour is used for initial segmentation of each time-point image individually, and 1.5 hour is used for 4D registration, and 0.5 hour for the 4D segmentation. The 2–3 iterations of 4D registration-segmentation is enough for a good segmentation. Overall, the proposed segmentation framework is able to achieve satisfactory segmentation results within a reasonable computational time. Note that more than 36 hours are needed for the FreeSurfer to process a typical serial images with 4–5 time-points.

To avoid enforcing any cortical-thickness constraint on the subcortical GM regions, we adopt the similar strategy in [19] to define a mask for the ventricular CSF and subcortical GM regions, where the cortical thickness constraint will not be imposed. In these subcortical regions, only local data fitting and atlas prior are employed to guide the segmentation.

Reported cortical thicknesses from post-mortem data in adults are in the range of 1.3–4.5 mm [12]–[14]. *In vivo* MR-based measurements from [35] were reported to have a mean thickness of 3.2 mm. Although, to the best of our knowledge, there are currently no studies measuring the physical cortical thickness, we conservatively set the acceptable range as 1–6.5 mm. The other weighting parameters 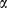, 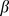 and 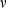 are set based on our experience.

In CLASSIC method, the follow-up images are rigidly aligned onto the baseline image, which may introduce bias, since the follow-up images will be interpolated. Instead, in this paper, to avoid bias, all the images are kept in their own space. In 4D registration, 4D-HAMMER registration algorithm was adopted in this paper. However, one limitation of 4D-HAMMER is to build a 4D template by repeating one specific 3D image as templates for different time-points, which may introduce bias in longitudinal data analysis. In our future work, we will use some more powerful registration methods, e.g., [36], to possibly avoid bias.

There are many definitions of cortical thickness [37]–[40]. For example, in [10], [40], the cortical thickness is defined as the minimum line integral on the probabilistic segmentation of GM. In [41], it is defined at each point as the length of the integral curve of the gradient field passing through that point. While, in this paper, the cortical thickness is defined as similar as [15], [42], which takes advantage of the level set function as a signed distance function. A comprehensive review on definitions of the cortical thickness can be found in [40]. This paper does not focus on how to measure the cortical thickness, but on how to ensure the temporal consistency of the cortical thickness. On the other hand, for other the definitions of cortical thickness in [10], [40], [41], we can also achieve the similar temporal consistent cortical thickness measurement using the same idea proposed in this paper.

Temporal cortical thickness constraint is introduced in this study to suppress the unwanted artificial variations between time-points. Too strong constraint may enforce the consistency between time-points and appear smoothing effects. In this paper, we tune the parameter for this constraint based on a set of training data. Experiments demonstrated that the proposed approach achieved results comparable to ground truth, and thus validates the setting of the parameter. The source code and software of the proposed method have been released in NITRC (http://www.nitrc.org/projects/abeat).
